# HDAC6 in Diseases of Cognition and of Neurons

**DOI:** 10.3390/cells10010012

**Published:** 2020-12-23

**Authors:** Patrizia LoPresti

**Affiliations:** Department of Psychology, University of Illinois at Chicago, 1007 West Harrison Street, Chicago, IL 60607, USA; patrizia.lopresti.22@gmail.com

**Keywords:** neurodegeneration, progressive multiple sclerosis, synapses, intracellular transport, aggregates, cognition

## Abstract

Central nervous system (CNS) neurodegenerative diseases are characterized by faulty intracellular transport, cognition, and aggregate regulation. Traditionally, neuroprotection exerted by histone deacetylase (HDAC) inhibitors (HDACi) has been attributed to the ability of this drug class to promote histone acetylation. However, HDAC6 in the healthy CNS functions via distinct mechanisms, due largely to its cytoplasmic localization. Indeed, in healthy neurons, cytoplasmic HDAC6 regulates the acetylation of a variety of non-histone proteins that are linked to separate functions, i.e., intracellular transport, neurotransmitter release, and aggregate formation. These three HDAC6 activities could work independently or in synergy. Of particular interest, HDAC6 targets the synaptic protein Bruchpilot and neurotransmitter release. In pathological conditions, HDAC6 becomes abundant in the nucleus, with deleterious consequences for transcription regulation and synapses. Thus, HDAC6 plays a leading role in neuronal health or dysfunction. Here, we review recent findings and novel conclusions on the role of HDAC6 in neurodegeneration. Selective studies with pan-HDACi are also included. We propose that an early alteration of HDAC6 undermines synaptic transmission, while altering transport and aggregation, eventually leading to neurodegeneration.

## 1. Introduction

Histone deacetylases (HDACs) play a central role in the epigenetic regulation of CNS function, with emphasis on development, neurodegenerative diseases, and various mental disorders [1,2,3]. Chromatin function is regulated by both HDACs and histone acetyltransferases (HATs). HDACs shape synaptic function and memory [4]. HDACs consist of eighteen HDAC isoforms. Eleven HDACs are zinc-dependent isoforms, including class I (HDACs 1/2/3/8), II (HDACs 4/5/6/7/9/10), and IV (HDAC 11). Seven HDACs (known as sirtuins) are nicotinamide adenine dinucleotide (NAD)-dependent and class III HDACs. Class I HDAC isoforms are largely nuclear whereas class II HDAC isoforms can be present in the nucleus and the cytoplasm. Class IV HDAC 11 is structurally diverse from class I and II HDAC isoforms [3]. Histone substrates and class I HDAC isoforms are considered central to memory regulation. Recent studies have placed the focus on non-histone targets of class II HDACs, with special emphasis on HDAC6 [5,6,7].

**Figure 1:**
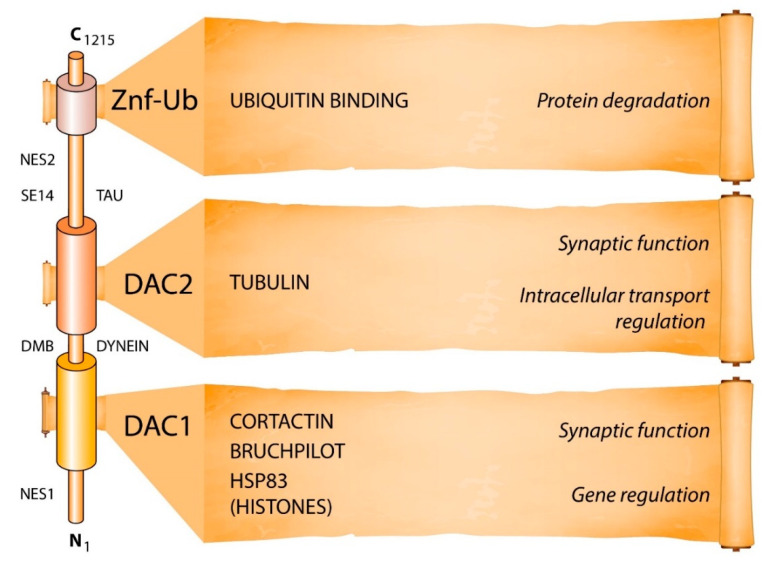
Domains and functions of HDAC6. HDAC6 is the largest protein of the HDAC family, with 1215 amino acid residues. ZnF-UB (zinc-finger ubiquitin binding) is a high-affinity ubiquitin-binding motif, and DAC (deacetylase) (1 and 2) is the domain with deacetylase activity. Targets for DAC1 and DAC2 are based on work largely done in Drosophila [13]. Additional targets include survivin, β-catenin, peroxiredoxin, and Miro1. NES = nuclear export sequences, DMB = dynein motor binding, SE14 = Ser-Glu tetradecapeptide repeating domain [9]. NES1 (Aa: 67–76), DAC1 (Aa: 87–447), DAC2 (Aa: 482–800), SE14 (Aa: 884–1022), NES2 (Aa: 1049–1058), ZnF-UB (Aa: 1131–1192) [9].

**Figure 2:**
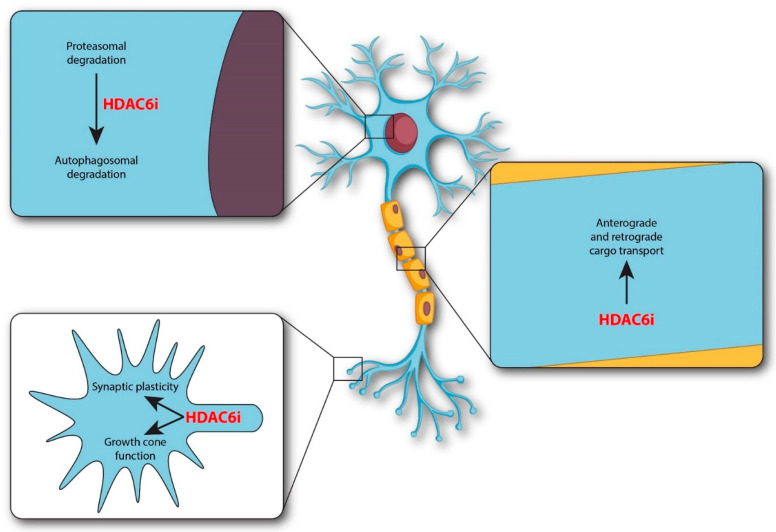
Cellular sites of HDAC6 inhibitors in neurodegenerative diseases. HDAC6 inhibitors (HDAC6i) regulate a variety of events including growth cone function, synaptic plasticity, transport, and autophagosomal degradation. The intracellular sites of regulation are indicated at the level of growth cone, processes, and soma.

HDAC6, a member of class IIb, localizes largely in the cytoplasm and is the only HDAC isoform with two tandem catalytic domains [8,9]. In addition, HDAC6 has a hydrolase-like zinc finger domain, that binds and transports polyubiquitinated protein aggregates, and a domain that regulates through protein–protein interactions the cytoskeleton protein tau, IIp45 (invasion inhibitory protein 45), and EGFR (epidermal growth factor receptor). Targets of its deacetylase activity include tubulin, cortactin, HSP (heat shock protein) 83/90, and Bruchpilot proteins (Figure 1). In various models of neurodegenerative diseases, pharmacological inhibition of HDAC6 restores alpha-tubulin acetylation and mitochondrial transport. In addition, HDAC6 inhibitors (HDAC6i) facilitate the degradation of protein aggregates and/or protection from neuronal oxidative stress (Figure 2) [9,10,11,12].

Tubacin is the original inhibitor of HDAC6, but additional inhibitors of this class are now available, including ACY-738 [7]. The zinc-binding residues in the catalytic domains of HDAC6 have been a target for the discovery of HDAC6i. HDAC6i have been developed via standard steps, which include pharmacophore generation, molecular docking, and molecular dynamics simulation. Pharmacophore models for new inhibitors of HDAC6 focus on a zinc-binding group (ZBG), a linker, and a cap. The molecular docking is aimed to show hydrogen bond interactions of the cap (in the HDAC6i) with the catalytic residues of HDAC6 [14]. Arylhydroxamate- based HDAC6i contain both a ZBG and a linker. However, the drugs in this group have shown toxicity and poor stability, which has significantly undermined their clinical use. In contrast, oxadiazole-based HDAC6i have led the way for additional HDAC6i with improved ADMET (absorption, distribution, metabolism, excretion, and toxicity) profiles. The list of compounds developed for these two classes of drugs is included in a previous review [8].

In the healthy brain, HDAC6 positively impacts synaptic function by its cytoplasmic localization [5], whereas in pathological conditions, HDAC6 undermines synaptic functions in part due to its abundance in the nucleus, with consequent transcription regulation and decreased expression of brain-derived neurotrophic factor (BDNF). For example, ApoE (Apolipoprotein E) and Aβ oligomers cause HDAC6 to largely translocate into the nucleus, where HDAC6 depresses BDNF transcription. The decrease in BDNF levels impairs memory [15]. Lee et al. [16] also describe the effects of HDAC6 in the prefrontal cortex (PFC) during acute stress regulation, highlighting the importance of HDAC6 for healthy higher brain functions. The PFC, a CNS region responsible for high-order cognitive functions, is highly influenced by stress. Acute stress affects PFC functions by potentiating glutamatergic transmission. HDAC6 inhibition or knockdown protects from the enhancement of glutamatergic transmission and glutamate receptor trafficking and from the effects of acute stress on synaptic functions [16].

## 2. Cognition Regulation and Neurodegeneration

Cognition and higher brain functions rely on precise protein–protein regulation at the synapses, which allows information among neurons to travel along functionally distinct regions of the CNS. In the synapses, selective proteins impact neurotransmission and memory regulation [13,17,18,19,20,21,22,23,24,25,26]. HDAC6 deacetylates Bruchpilot (flies), which enhances neurotransmitter release [27]. Although the specific mammalian protein regulated by HDAC6 is unknown, important candidates include CAST (cytomatrix at the active zone-associated structural protein)/ELK proteins, which are the equivalent to Bruchpilot. CAST/ELK proteins interact with a set of proteins to regulate synaptic transmission. These proteins include RIM (Rab-interacting molecule), Munc13-1, Bassoon (Bsn), and Piccolo. CAST/ELKS in the hippocampus binds Bsn to regulate short-term plasticity [13,17,18,19,20,21,22,23,24,25,26,27]. Alterations of acetylation levels of selected proteins at the synapses would change both cognition and protein precipitation.

Of particular interest, Bsn aggregates have been found in experimental autoimmune encephalomyelitis (EAE) mice and in multiple sclerosis (MS) patients [28]. Bsn is a scaffolding protein, and CAST is known to bind the Bsn protein. Thus, future studies are needed to define how early alterations in HDAC6 impact the development of Bsn precipitates and whether the inhibition of HDAC6 would prevent the formation of Bsn precipitates. Determining which mammalian protein at the synapse is targeted by HDAC6 would be an area of intense research in order to understand memory and aggregate regulation and neurodegeneration.

HDAC6 is highly conserved from flies to mammals. Although mammals do not have a direct homolog of Bruchpilot, it is conceivable that HDAC6 targets selective (mammalian) proteins at the synapses [13,17,18,19,20,21,22,23,24,25,26,27]. Because the human version of Bruchpilot targeted by HDAC6 has to be identified, it is essential to identify the protein(s) responsible for synaptic plasticity, memory, and neurodegeneration in mammals. Most likely, HDAC6 modulates the acetylation of the short cytoplasmic tail of CAST and/or proteins that bind CAST. Proper acetylation is the result of acetylation and deacetylation. Acetylation is mediated by elongator protein 3 (ELP3), whereas deacetylation is mediated by HDAC6. The work by Miśkiewicz et al. [27] has elucidated memory regulation by acetylation. Acetylation of lysine in the cytoplasmic domain of Bruchpilot (flies) is critical for synaptic plasticity. In Drosophila, acetylation levels in the long C-terminal tail of Bruchpilot calibrate the number of vesicles to be released and synaptic transmission [13,17,18,19,20,21,22,23,24,25,26,27,29]. In contrast, hypoacetylation would cause the release of excess vesicles with aberrant neurotransmission and excitotoxity [29]. Deleterious effects would also occur with too much acetylation and insufficient numbers of vesicles. Thus, Bruchpilot acetylation calibrates the release of the correct number of neurotransmitters. It is imperative to identify the one or more mammalian proteins serving a similar function. These selective proteins would be key for understanding both memory and neurodegeneration.

Hypoacetylation is present during neurodegeneration [2]. An altered excitatory (E)-inhibitory (I) balance of synaptic transmission occurs early during neurodegenerative diseases. Such alterations prime cognition deficits. Indeed, in ALS (amyotrophic lateral sclerosis), the E-I balance is altered earlier during the disease [30], whereas ALS-specific cognitive and behavior changes have been associated with advanced stages of ALS [31]. TDP-43 (TAR DNA binding protein 43) normally resides in the nucleus and binds DNA and RNA to regulate RNA splicing and influence transcript stability, transport, and translation [32,33].The functional relationship between TDP-43 and HDAC6 is important in the context of ALS. TDP-43 upregulates HDAC6 expression. In summary, deregulation of HDAC6 causes alterations in deacetylation, E/I balance, memory, and protein processing. Of interest, Rossaert et al. [34], by using HDAC6i, showed restoration of histone acetylation and improvement in disease course and metabolic abnormalities in a FUS (fused in sarcoma) mouse model of ALS.

Cognitive deficits cover quite a wide range of CNS diseases, ranging from neuropsychiatric disorders in children and adults to neurodegenerative diseases [2,35]. Studies have shown that behavior alterations are present quite early during MS. At the very early stage of the disease, a silent, i.e., subclinical, decline in neuronal function takes place [36]. Such a decline would precede neurodegeneration. Recent studies in both MS and EAE have shown that subclinical, early events at disease onset might shape neurodegeneration and progressive MS [36]. A key goal is to determine the molecular and cellular basis of such early alterations at the synapses in order to prevent the progressive neurodegeneration evident in the secondary progressive form and in the primary form of the disease [36,37].

We have shown that when given acutely, ACY-738, the selective HDAC6 inhibitor, has an effect on memory in a fashion that is sensitive to disease severity [37]. In particular, when various concentrations of EAE-inducing reagents are used, EAE C57BL/6 mice immunized with higher amounts do not display increased short-term memory following ACY-738 treatment. This lack of response (to the drug) at 10 days post-immunization (dpi) (before any mobility impairment is apparent) most likely reveals an early defect at the level of the synapses that would lead to neurodegeneration. Presumably, the alterations of memory regulation result from an inflammatory environment having an effect on selective cytoskeleton proteins and receptors that are involved in memory regulation [37].

HDAC6 is a multidomain protein, with domains that are linked to transport regulation, aggregate formation and cognition (Figure 1 and Figure 2). HDAC6 deacetylates tubulin and Bruchpilot for transport and synaptic regulation, respectively, while at the same time, HDAC6 binds ubiquitinated proteins for protein aggregation regulation [5,11,13,27]. Demyelinating diseases often feature alterations in transport regulation and in aggregate formation, together with cognitive decline [36,37,38,39,40,41]. Future studies would need to determine whether the complex sets of pathologies present during MS derive from a primary defect occurring at the level of HDAC6, occurring quite early during the disease.

Compelling evidence indicates that neurocognition is affected before disease onset in EAE mice. Furthermore, cognitive deficit manifests following a pattern of subclinical cognitive decline, which has been found in both MS patients and in EAE mice [36].

The study by Liu et al. [42] investigated the effects of HDAC6 on functional and pathological changes in amyloid beta (Aβ)-induced cognitive dysfunction in rats. This study found that HDAC6 may not only lead to the deterioration of learning and memory abilities but may also elevate levels of Aβ and Tau phosphorylation, both having deleterious effects on the disease

Vascular dementia (VD) is a common dementia disease, second in the world only to Alzheimer’s disease (AD) [43]. Donepezil, used to treat mild to moderate AD, has been shown to treat cognitive impairment and memory deficits caused by VD. Jian et al. [43] showed that donepezil treatment significantly improved cognitive performance. Furthermore, donepezil treatment significantly attenuated neurodegeneration and restored synapse dendritic spine density in the cortex and hippocampus. Donepezil attenuates neurodegeneration by correcting mis-localization of HDAC6 and increasing BDNF expression.

In the brains of patients with AD, highly phosphorylated pathological Tau accumulates and causes neuronal loss, synaptic dysfunction, and cognitive decline. Choi et al. [44] found that HDAC6 CDK-504 inhibitor degrades pathological Tau because acetylated Tau recruits chaperone proteins, leading to the degradation of pathological Tau through the proteasomal pathway. Overall, the study shows that CDK-504 ameliorates synaptic and cognitive anomalies, most likely by activating the chaperone machinery and degrading pathological Tau. Similar beneficial effects in mouse models of AD have been obtained with a variety of HDAC6i [8,45,46,47,48,49,50,51,52].

An important area for intervention is in chemotherapy-related cognitive impairment occurring as a long-term side effect during cancer treatment. Wang et al. [53] examined the effect of the HDAC6 inhibitor, ACY-1215 (Ricolinostat), on cisplatin-induced brain damage and cognitive deficits in mice. The study showed that ACY-1215 ameliorated behavioral deficits and dendritic spine loss and increased synaptic density in cisplatin-treated mice. ACY-1215 enhanced α-tubulin acetylation in the hippocampus of cisplatin-treated mice with diminished impairment of mitochondrial transport and mitochondrial dysfunction. Thus, HDAC6 inhibition improves cisplatin-induced cognitive deficits by reversing mitochondrial and synaptic functional impairments.

An area of great interest is the treatment of human immunodeficiency virus (HIV)-associated neurocognitive disorders. The neurological damage observed in HIV-positive subjects can be experimentally reproduced by the HIV envelope glycoprotein (gp)120. gp120 binds to neuronal microtubules and decreases the level of tubulin acetylation, with resulting impairment of axonal transport [54,55]. Wenzel et al. [54] showed that the selective HDAC6i tubacin and ACY-1215 prevented gp120-mediated deacetylation of tubulin and inhibited the ability of gp120 to decrease axonal transport. Indeed, the study showed that gp120 decreases the velocity of BDNF transport, which is restored to baseline levels when neurons are exposed to HDAC6i [55].

Animal studies demonstrated that multiple exposures to sevoflurane (used as an inhalational anesthetic) during the postnatal period impaired synaptogenesis, resulting in cognitive deficits [56]. Multiple sevoflurane exposures enhanced HDAC6 expression and activity in the hippocampi of the developing brain. Tubastatin A ameliorated sevoflurane-induced decreases in synaptophysin and PSD95 (postsynaptic density protein 95) expression, improving synaptic ultrastructural damage and cognitive deficits. In conclusion, HDAC6 is involved in the developmental neurotoxicity caused by multiple sevoflurane exposures and HDAC6 inhibition prevents neuronal damage [56].

While work in EAE mice showed behavioral alterations as early as the first week postimmunization, precipitates of the synaptic protein Bsn were detected at 16 dpi [28]. Understanding the functional relationship between cognition and aggregate regulation could provide new insights into mechanisms of neurodegeneration. We propose that an alteration of HDAC6 early during the disease causes both memory dysfunction and precipitates, since HDAC6 has functional domains for both processes.

Epigenetics and histone modifications are crucial events in diseases of cognition, neurodegeneration, and development. These modifications have been also extensively studied in MS and play an important role in both the initiation and development of MS [57,58,59].

Although genetic susceptibility to MS is not completely understood, HLA-DR (human leukocyte antigen—DR isotype)-specific expression has an impact on the disease. Of interest, HLA-DR is regulated by HDAC1. Since, in pathological conditions, HDAC6 translocates to the nucleus, the functional relationship with nuclear HDAC must be considered in the context of epigenetic regulation during the inflammation and neurodegeneration that occur in MS. Of great interest, HDAC gene variants predict brain volume changes in multiple sclerosis [60]. In this context, future studies are needed to evaluate in detail the role of HDAC6.

Therapeutic approaches that promote neuroprotection counter CNS neurodegenerative diseases. Neurodegeneration in MS defines poor clinical outcome and quality of life. In MS, neuroprotection requires the regulation of both autoimmune demyelination and inflammation [61,62,63]. Autoimmune demyelination starts as antigen-presenting cells (APCs) mature and migrate into the lymph nodes, where they present the antigen to T cells. During activation, T cells differentiate into mature effector CD4^+^ T cell subsets (Th1, Th2, Th17, Treg). T helper 1, 2, and 17, and Treg cells then translocate into the CNS, where they are reactivated by resident APCs like microglia [63]. Additional inflammatory cells are then recruited to the CNS, which further enhances inflammation and tissue damage.

Several studies have investigated the functional consequences of HDACi on various cell types having a role during MS disease [64]. Treg cells are known to be protective, whereas Th1/Th17 cells are deleterious for the disease. HDACi facilitate Treg cells. In contrast, HDACi inhibit Th1/Th17 cells. In addition, cytokines released by T cells modulate synaptic functions. HDACi inhibit CD4^+^ cell proliferation and Interferon gamma production. Such inhibitors also suppress proinflammatory Interleukin-2 cytokine released from Th1 cells, while HDACi such as Trichostatin A and suberoylanilide hydroxamic acid (SAHA) have a positive effect on Treg cells [64]. In summary, HDACi modulate MS disease, through combined actions on distinct cells, with the resulting effect of improving protection while diminishing cell damage.

Of particular importance, the effects of HDACi on oligodendrocytes (OLGs) must be viewed with caution, considering possible differences in the effects of HDACi in early vs. late phases of the disease. High levels of histone acetylation exist in undifferentiated oligodendrocyte precursor cells (OPCs). Histone3 acetylation in OLG inhibits OLG differentiation. In contrast, histone deacetylation favors OLG differentiation and myelination [65,66,67]. HDAC6 also regulates acetylated Tau in OLGs. Tau participates in many aspects of OLG biology, including myelin formation, myelin integrity, and myelin repair [68,69,70]. The relationship between HDACi and inflammation is a complex one. HDACi can affect astrocytes and microglia [71]. HDAC inhibition prevents white matter injury by modulating microglia/macrophages [71]. Work by Faraco et al. [72,73] showed that HDACi such as SAHA and Givinostat induce a dramatic increase in histone acetylation without causing cytotoxicity of cultured mouse glial cells. In addition, these two compounds inhibit the expression of proinflammatory mediators produced by lipopolysaccharide-challenged glial cultures, while potentiating in vitro immunosuppression triggered by dexamethasone. It seems that mouse glial cells have ongoing HDAC activity, and HDACi inhibit the neuroinflammatory response, secondary to direct impairment of the transcriptional machinery [72,73]. Additional work is required to understand the specific role of HDAC6 in MS pathogenesis and OLG biology.

Work by Sun et al. [74] showed that the loss of HDAC11 ameliorates clinical symptoms in a multiple sclerosis mouse model. In particular, the study showed that loss of HDAC11 significantly reduces disease severity and spinal cord demyelination during the post-acute phase of EAE. The absence of HDAC11 reduced immune cell infiltration into the CNS, with decreased monocytes and myeloid dendritic cells during the chronic progressive phase of the disease. Thus, the authors suggest the use of HDAC11-specific inhibitors to treat chronic progressive MS [74]. Since our work showed that a selective HDAC6 inhibitor delays disease onset and decreases disease severity [37], HDAC11 appears to act at a later time. Thus, future studies using a combination of pharmacological and genetic approaches are needed to determine the role of each specific HDAC isoform at various times during MS, and how the disease may benefit from approaches that include inhibitors for both HDAC11 and HDAC6 isoforms.

HDACi are also emerging as neuroprotective agents during various diseases, including diseases that follow an ischemic insult. Faraco et al. [75] investigated the effect of the HDAC inhibitor SAHA on histone acetylation in control and ischemic mouse brains. Ischemic insult was obtained following middle cerebral artery occlusion lasting 6 h. Brain histone H3 acetylation was constitutively present at specific lysine residues in neurons and astrocytes, whereas in ischemic brain tissue, histone H3 acetylation levels were drastically decreased. Treatment with SAHA increased histone H3 acetylation, preventing histone deacetylation in the ischemic brain. As a result of this drug treatment, expression of the neuroprotective proteins Hsp70 and Bcl-2 (B-cell lymphoma 2) increased. This work demonstrates that pharmacological inhibition of HDACs promotes the expression of neuroprotective proteins, suggesting that HDACi may be beneficial for stroke therapy [76]. Further, Wang et al. [77] showed that the HDAC6 inhibitor Tubastatin A alleviates stroke-induced brain infarction and functional deficits.

Acetylation homeostasis is believed to be a key regulator of both immune cell activation and neuronal survival. Of note, HDACi with both anti-inflammatory and neuroprotective properties have been identified [72]. Efficacy of HDACi in experimental models of MS has been reported consistently [37,72]. In other neurodegenerative diseases such as ALS, AD, SMA (Spinal muscular atrophy), HD (Huntington’s disease), and PD (Parkinson’s disease), histone acetylation homeostasis is greatly impaired, shifting toward a state of hypoacetylation [2]. Indeed, histone hyperacetylation produced by direct inhibition of HDACs leads to neuroprotection [78,79].

## 3. Intracellular Transport and Neurodegeneration

HDAC6 binds and deacetylates tubulin and thereby regulates intracellular transport. Neuronal transport defects in ALS are restored with HDAC6 inhibitors [34]. Transport defects are also detected in EAE as early as two days following onset of mobility impairment [38].

Guo et al. [80] showed that mitochondrial Rho GTPase 1 (Miro1) is required for axonal transport of mitochondria into Drosophila synapses. Miro1 facilitates mitochondrial transport by attaching the mitochondria to the motor/adaptor complex [10,80,81].

With regard to a role of HDAC6 in transport regulation, Kalinski et al. [10], interestingly, showed that HDAC6 deacetylates Miro1, with resulting inhibition of mitochondria transport and axon growth. In contrast, HDAC6i increase transport and axon growth via increased levels of acetylated Miro1. Axon growth is inhibited by myelin largely via the Rho/Rock (Rho-associated protein kinase) pathway. Kalinski et al. [10] showed that increased levels of acetylated Miro with HDAC6i compensate for myelin/MAG (Myelin-associated glycoprotein) induced inhibition of axon growth.

HDAC6i are also beneficial in Charcot-Marie-Tooth type 2D (CMT2D), a disease with motor and sensory axonal peripheral neuropathy [82,83,84,85,86,87,88]. Mutations in the glycyl-tRNA synthetase gene cause CMT2D. Interaction of HDAC6 with mutated glycyl-t RNA synthetase is deleterious for intracellular transport regulation. In contrast, such interaction is blocked by the HDAC6 inhibitor Tubastatin A, with positive effects on axonal transport [82,83,84,85,86,87,88].

Rett syndrome (RTT) is a developmental disorder with an alteration in the transport of BDNF. RTT is a rare genetic neurological and developmental disorder caused by loss-of-function mutations in the transcriptional modulator methyl-CpG-binding protein 2 (MECP2) [89]. One of the most prominent gene targets of MECP2 is BDNF, a potent modulator of activity-dependent synaptic development, function, and plasticity. In RTT, BDNF signaling has been shown to be impaired. The selective HDAC6 inhibitor Tubastatin A increases α–tubulin acetylation, leading to increased anterograde and retrograde transport of BDNF in Mecp2 knockout neurons [89]. Tubastatin A also restores activity-dependent BDNF release from MECP2 knockout neurons to levels comparable to those of wild-type neurons. These findings demonstrate that HDAC6i can be used as a potential pharmacological strategy to reverse cellular and synaptic impairments in RTT resulting from impaired BDNF signaling [8,89].

How intracellular signaling affects HDAC6 is of paramount importance in order to implement effective approaches that modulate HDAC6 and intracellular transport. Cao et al. [90] showed that MAPK (mitogen-activated protein kinase) regulates HDAC6 localization and phosphorylation, in addition to having positive effects on tyrosinated tubulin and microtubule (MT) stability.

## 4. Aggregates and Neurodegeneration

The ability of HDAC6 to bind and regulate ubiquitinated proteins and the proteasome machinery sets HDAC6 in the context of important events of protein homeostasis [9]. In neurodegenerative ALS disease, stress granules become associated with HDAC6 instead of with TDP-43 (TAR DNA binding protein 43-kDa), resulting in decreased clearance of this toxic nucleic acid-binding protein and deleterious consequences [91,92]. Mutated TDP-43 (a DNA/RNA binding protein) increases HDAC6 expression and decreases Bruchpilot acetylation. Bruchpilot, a target of HDAC6, regulates neurotransmission in Drosophila. Defects present with mutated TDP-43 are similar to those seen upon increased HDAC6 expression and contrast those present in HDAC6 null mutants. These defects include, among others, changes in neurotransmission [91].

The study of Kawaguchi et al. [92] shows that HDAC6 is a component of the aggresome and that HDAC6 can bind both polyubiquitinated misfolded proteins and dynein motors.

In demyelinating inflammatory diseases such as EAE and MS, the synaptic protein Bsn accumulates in the neuronal soma [28]. Bsn precipitates are deleterious for neuronal survival, and clearance of this protein with pharmacological proteasome activation decreases Bsn load while increasing neuronal survival. It would be worth testing, in future studies, whether HDAC6 inhibitors are able to prevent Bsn precipitates.

Functional connections have also been found between HDAC6 activity and stress granule dynamics. HDAC6 binds Ras GTPase-activating protein-binding protein 1 (G3BP-1), a stress granule nucleator. The catalytically inactive MAP kinase phosphatase (MK-STYX) interacts with the stress granule nucleator G3BP-1 to decrease stress granule formation. Since MK-STYX is a signaling molecule along HDAC6 activity, future studies should explore in more detail the relationship between HDAC6i and stress granule regulation [90].

Protein aggregates are a common feature of CNS neurodegenerative disease. Bsn aggregates have been found in MS patients and in the animal model of MS [28], whereas TDP-43 harboring mutations that cause ALS are believed to be responsible for TDP-43 aggregates and decreased Bruchpilot acetylation [91,92]. Yan et al. [93] show that pharmacological inhibition of HDAC6 with Tubastatin A attenuates inflammatory response and protects dopaminergic neurons in experimental models of PD [93,94,95,96].

## 5. Overview

Deregulation of selected HDAC isoforms has been connected to various CNS diseases, including degenerative, developmental, and psychiatry disorders [1,2]. These diseases include ALS, AD, PD, CMT2D, RTT, Fragile X Syndrome (FXS), Rubinstein–Taybi, depression, and schizophrenia. In addition, deregulation of HDAC activity has been found in stroke and traumatic brain injury [75,97]. All these diseases have disturbance of cognition, mood, and experience-dependent plasticity. During these diseases, the epigenetic basis of the modifications includes decreased histone acetylation and increased DNA and H3K4 methylation.

HDAC6 targets alpha tubulin and beta catenin [5]. Beta catenin has the interesting feature of binding to PDZ (postsynaptic density 95, PSD-85; Discs large, Dlg; Zonula occludens-1, ZO-1)-containing proteins, known largely for their scaffolding functions. HDAC6i increase neuronal survival and protect from axonal degeneration, while decreasing disease progression and improving memory. HDAC6i improve also axonal transport, α-tubulin acetylation, MT defects, and protein aggregates [78,79].

In the context of cognitive defects, FXS has various developmental anomalies including learning disability, cognitive impairment, and behavioral pathologies. FXS is caused by transcriptional silencing of the fragile X mental retardation (FMR) gene with the corresponding protein (FMRP) having a role in mRNA trafficking and dendritic translation of proteins involved in learning and memory. Of interest, the HDAC6 inhibitor SW-100 improves the phenotype in animal models of FXS [98].

HDAC6 inhibition also induces mitochondrial fusion and autophagic flux and reduces diffuse mutant huntingtin in striatal neurons [99]

ALS is a neurodegenerative disease of motor neurons with dominant mutations in two related RNA-binding proteins, TDP-43 and FUS/TLS (fused in sarcoma/translocated in liposarcoma) that cause a subset of ALS. FUS/TLS is a multifunctional DNA-/RNA-binding protein and TDP-43 interacts with FUS/TLS to form a complex in mammalian cells. RNAi silencing of either TDP-43 or FUS/TLS reduces HDAC6 mRNA expression [91].

Table 1 shows the half maximal inhibitory concentration (IC50) and the selectivity over HDAC 1, 2, and 3 for each HDAC6 inhibitor. The HDAC6i Tubastatin A, SW-100, and MPTOG211 are 1,000-fold more selective over HDAC 1, 2, and 3.

**Figure 3:**
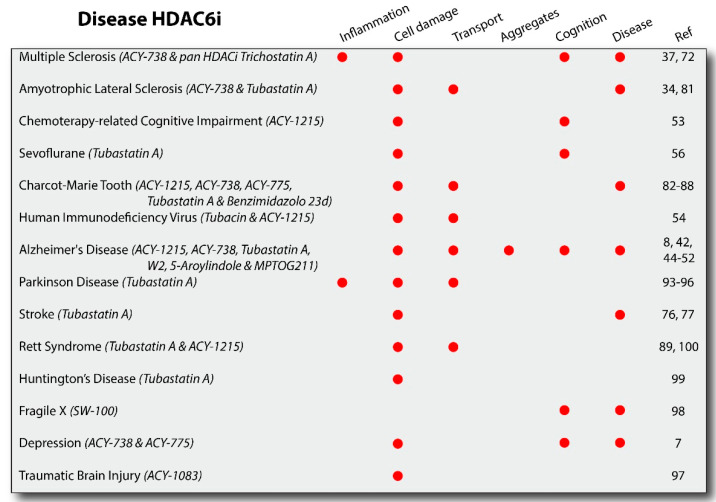
HDAC6 inhibitors in neurodegenerative diseases. HDAC6 inhibitors (HDAC6i) have been studied in several diseases (e.g., degenerative, developmental, and following toxins/infection) and have been shown to improve a combination of pathologies including inflammation, cell damage, transport regulation, aggregation, cognition, and disease course. The results shown in this figure reflect studies done with the drugs as indicated. As additional HDAC6i are developed and more studies are performed, additional results could add insight into the overall benefits of this class of drugs. All the drugs indicated are HDAC6i with the exception of Trichostatin A, an inhibitor for class I and II HDAC isoforms.

Figure 3 shows the results obtained with specific HDAC6i used in in vivo models of the specific disease, as indicated. Selective studies were in cell culture models for ALS, RTT, AD, and HD diseases and in induced pluripotent stem cells (iPSCs) derived from patients with RTT [100]. Additional information for each HDAC6 inhibitor can be found in the Supplementary Materials.

## 6. Concluding Remarks and Future Perspectives

Understanding CNS degeneration remains central for effective therapeutic options. Alterations of synaptic functions have been found in MS and its animal model, EAE, using electrophysiological and behavioral approaches. More recently, a functional defect at the synapses has been shown in EAE mice at 10 dpi before any motor defects are detected [37]. EAE mice immunized with a high dose of EAE-inducing reagents and with a chronic disease course do not have an increased short-term memory in response to the HDAC6 inhibitor ACY-738. In contrast, EAE mice immunized with lower doses and having a milder disease course retain the ability to respond to the drug with an increase in short-term memory [37]. These data, to our knowledge, were the first to directly indicate that disease activity calibrates synaptic function. Thus, an inability of HDAC6 inhibitor to work at the very beginning of disease might indicate synaptic alterations, which could prime neurodegeneration [36,37]. How changes in HDAC6 account for altered behaviors and electrophysiological properties previously described in EAE mice will require additional studies.

The challenge will be to determine in MS the molecular events linking HDAC6 to alterations in short-term memory, Bsn precipitates, and transport regulation. A previous study showed that HDAC1 localization changes as it translocates from the nucleus to the cytoplasm under demyelinating-like conditions [101]. Whether the alterations of HDAC1 and HDAC6 compound into neurodegeneration or are independent alterations with separate timing of occurrence will have to be addressed in future studies. In conclusion, understanding how disease activity alters functional synaptic circuits will provide a new understanding of neurodegeneration in MS.

## 7. Highlights

Neurodegeneration during MS is devastating. Understanding the cause of neurodegeneration is imperative for potential treatments able to modify the neurodegeneration and the course of the disease.

Histone deacetylase inhibitors are important for the treatment of CNS degenerative diseases [78,79]. Most HDAC isoforms localize to the nucleus and regulate transcription. However, HDAC6 localizes in the cytoplasm and largely regulates synaptic function. Analysis of HDAC6 molecules has revealed multifunctional domains that affect multiple events known to be altered during neurodegenerative disease. HDAC6 domains regulate intracellular transport, synaptic function, and protein processing.

## 8. Outstanding Questions

What are the respective contributions of distinct HDAC6 domains for neurodegeneration? Can these distinct functional entities be clearly identified or do contributions of the different domains rely on their dynamic interplay?

Which are the underlying mechanisms and signaling pathways for each domain?

Are possible alterations independent from each other? Which alteration emerges first to prime neurodegeneration? Can tracing and interfering with the earliest alterations protect from neurodegenerative events?

Is cognition alteration a quite early event, followed by the secondary alterations responsible for neurodegeneration?

## Figures and Tables

**Table 1:** HDAC6 inhibitors and selectivity over HDAC 1, 2, and 3. The half maximal inhibitory concentration (IC50) is indicated for each HDAC6 inhibitor. The selectivity over HDAC 1, 2, and 3 is indicated for each drug. The selectivity index is the ratio of each HDAC IC50 vs. HDAC6 IC50.

HDAC6i	IC50 HDAC6	HDAC1/6	HDAC2/6	HDAC3/6
**ACY-738**	1.7 nM	55-fold	75-fold	128-fold
**ACY-1215**	4.7 nM	12-fold	10-fold	11-fold
**Tubacin**	4.0 nM	350-fold	>1000-fold	318-fold
**Tubastatin A**	4.4 nM	>1000-fold	>1000-fold	>1000-fold
**SW-100**	2.3 nM	>1000-fold	>1000-fold	>1000-fold
**ACY-775**	7.5 nM	283-fold	347-fold	>1000-fold
**ACY-1083**	3.0 nM	>260-fold	>260-fold	>260-fold
**MPTOG211**	0.29 nM	>1000-fold	>1000-fold	>1000-fold
**W-2**	21 nM	153-fold	60-fold	63-fold
**5-Aroylindole 6**	3.92 nM	559-fold	145-fold	>1000-fold

## Supplementary Materials

The following are available online at https://www.mdpi.com/2073-4409/10/1/12/s1.
