# Supplementary material for: HDAC6 in Diseases of Cognition and of Neurons
Source: Cells. 2020 Dec 23;10(1):12. doi: 10.3390/cells10010012 (PMC7822434; doi:10.3390/cells10010012)
Supplement: Supplementary file 1 [file cells-10-00012-s001.pdf]

Supplementary Materials

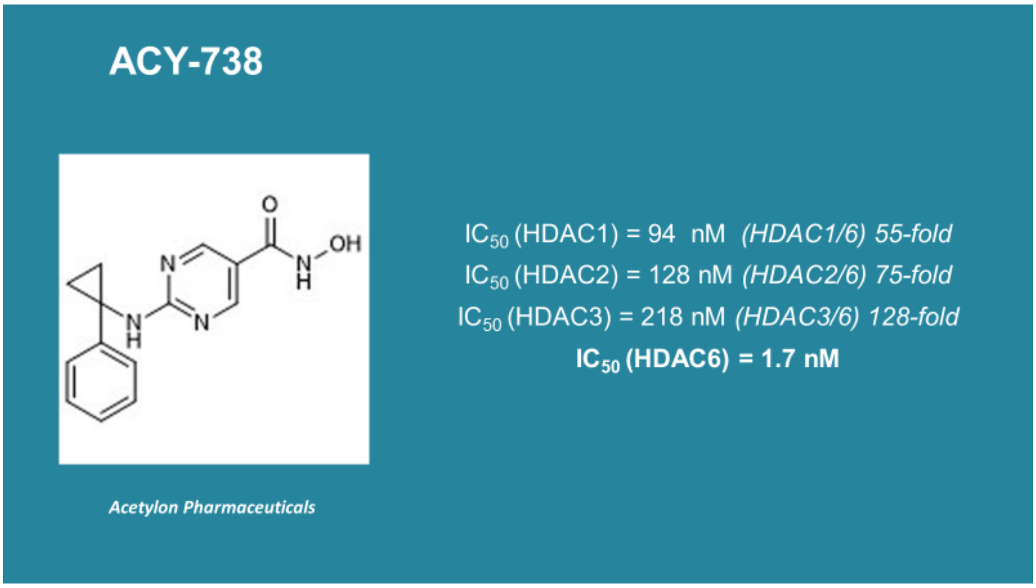

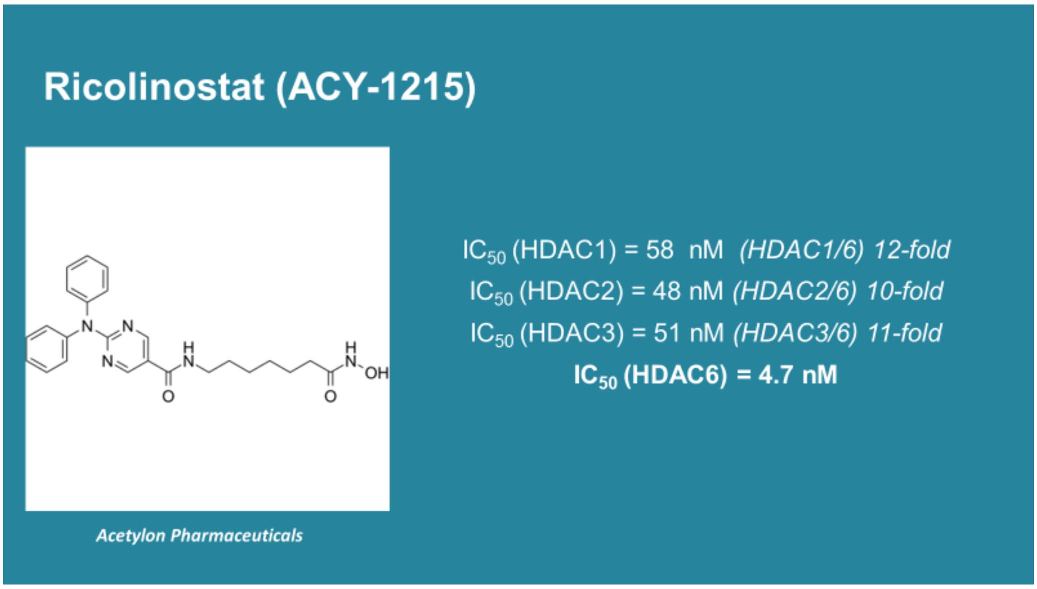

Tubacin

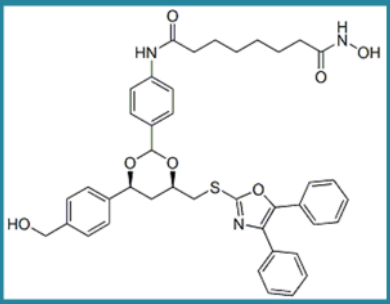

IC_{50} (HDAC1) = 1,400 nM *(HDAC1/6) 350-fold*
IC_{50} (HDAC2) = 6,270 nM *(HDAC2/6) >1000-fold*
IC_{50} (HDAC3) = 1,270 nM *(HDAC3/6) 318-fold*
IC_{50} (HDAC6) = 4 nM

Dr. Stuart L. Schreiber

Tubastatin A

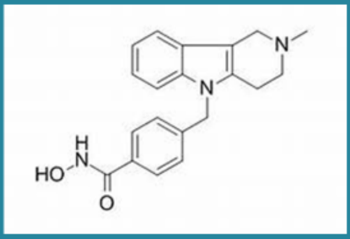

IC_{50} (HDAC1) = 8,100 nM *(HDAC1/6) > 1000-fold*
IC_{50} (HDAC2) = 18,600 nM *(HDAC2/6) > 1000-fold*
IC_{50} (HDAC3) = 7,6000 nM *(HDAC3/6) > 1000fold*
IC_{50} (HDAC6) = 4.4 nM

Dr. Alan P. Kozikowski group

SW-100

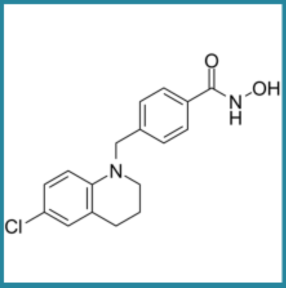

IC_{50} (HDAC1) = 5,230 nM *(HDAC1/6) >1000-fold*
IC_{50} (HDAC2) = 32,800 nM *(HDAC2/6) >1000-fold*
IC_{50} (HDAC3) = 29,500 nM *(HDAC3/6) >1000fold*
IC_{50} (HDAC6) = 2.3 nM

Dr. Alan P. Kozikowski group

ACY-775

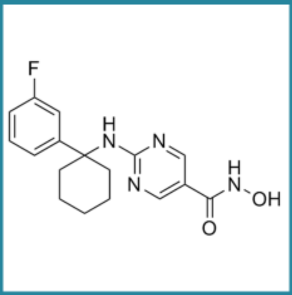

Acetylon Pharmaceuticals

IC_{50} (HDAC1) = 2,123 nM *(HDAC1/6) 283-fold*
IC_{50} (HDAC2) = 2,570 nM *(HDAC2/6) 343-fold*
IC_{50} (HDAC3) = 11,223 nM *(HDAC3/6) >1000-fold*
IC_{50} (HDAC6) = 7.5 nM.

ACY-1083

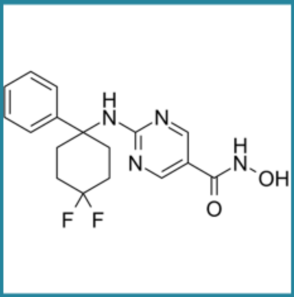

Acetylon Pharmaceuticals

HDAC1/6 > 260-fold
HDAC2/6 >260-fold
HDAC3/6 > 260-fold
IC_{50} (HDAC6) = 3 nM

MPTOG211

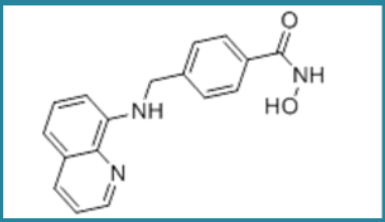

IC_{50} (HDAC1) = 9,550 nM*(HDAC1/6) >1,000-fold*
IC_{50} (HDAC2) = 12,500 nM *(HDAC2/6) >1,000-fold*
IC_{50} (HDAC3) = 7,750 nM *(HDAC3/6) >1,000-fold*
IC_{50} (HDAC6) = 0.29 nM

Dr. Jing-Ping Liou group

Legend. HDAC6 inhibitors and selectivity over HDAC 1, 2, and 3. The half maximal inhibitory concentration (IC50) is indicated for each HDAC6 inhibitor. The selectivity over HDAC 1, 2, and 3 is indicated for each drug.

Additional HDAC6 inhibitors

HDAC6 inhibitor W-2 from Dr. Alan P. Kozikowski group, IC50 21 nM.

HDAC1/6 153 fold, HDAC2/6 59.5 fold, HDAC3/6 62.9 fold.

HDAC6 inhibitor 5-Aroylindole 6 from Dr. Jing-Ping Liou group, IC50 3.92 nM.

HDAC1/6 558.7 fold, HDAC2/6 144.9 fold, HDAC3/6 > 1000 fold.

References

1. Haggarty, S.J.; Koeller, K.M.; Wong, J.C.; Grozinger, C.M.; Schreiber, S.L. Domain-selective small-molecule inhibitor of histone deacetylase 6 (HDAC6)-mediated tubulin deacetylation. *Proc. Natl. Acad. Sci.* **2003**, *100*, 4389–4394, doi:10.1073/pnas.0430973100.
2. Jochems, J.; Boulden, J.; Lee, B.G.; A Blendy, J.; Jarpe, M.; Mazitschek, R.; Van Duzer, J.H.; Jones, S.; Berton, O. Antidepressant-Like Properties of Novel HDAC6-Selective Inhibitors with Improved Brain Bioavailability. *Neuropsychopharmacol.* **2014**, *39*, 389–400, doi:10.1038/npp.2013.207.
3. Pulya, S.; Amin, S.A.; Adhikari, N.; Biswas, S.; Jha, T.; Ghosh, B. HDAC6 as privileged target in drug discovery: A perspective. *Pharmacol. Res.* **2020**, *105274*, 105274, doi:10.1016/j.phrs.2020.105274.
4. Shen, S.; Kozikowski, A.P. A patent review of histone deacetylase 6 inhibitors in neurodegenerative diseases (2014-2019). *Expert Opin. Ther. Patents* **2019**, *30*, 121–136, doi:10.1080/13543776.2019.1708901.
5. Nikolian, V.C.; Dennahy, I.S.; Weykamp, M.; Williams, A.M.; Bhatti, U.F.; Eidy, H.; Ghandour, M.H.; Chtraklin, K.; Li, Y.; Alam, H.B. Isoform 6–selective histone deacetylase inhibition reduces lesion size and brain swelling following traumatic brain injury and hemorrhagic shock. *J. Trauma Acute Care Surg.* **2019**, *86*, 232–239, doi:10.1097/ta.0000000000002119.
